# Diagnostic performance of line-immunoassay based algorithms for incident HIV-1 infection

**DOI:** 10.1186/1471-2334-12-88

**Published:** 2012-04-12

**Authors:** Jörg Schüpbach, Leslie R Bisset, Martin D Gebhardt, Stephan Regenass, Philippe Bürgisser, Meri Gorgievski, Thomas Klimkait, Corinne Andreutti, Gladys Martinetti, Christoph Niederhauser, Sabine Yerly, Stefan Pfister, Detlev Schultze, Marcel Brandenberger, Franziska Schöni-Affolter, Alexandra U Scherrer, Huldrych F Günthard

**Affiliations:** 1 Swiss National Center for Retroviruses, Institute of Medical Virology, University of Zurich, Winterthurerstrasse 190, Zurich CH-8057, Switzerland; 2 Swiss Federal Office of Public Health, Berne CH-3003, Switzerland; 3 Clinic for Immunology, University Hospital, Zurich CH-8044, Switzerland; 4 Service d'Immunologie et d'Allergie, University Hospital, Lausanne CH-1011, Switzerland; 5 Institute of Infectious Diseases, University of Berne, Berne CH-3010, Switzerland; 6 Institute for Medical Microbiology, University of Basel, Basel CH-4003, Switzerland; 7 Clinique de la Source, Laboratoire, Lausanne CH-1004, Switzerland; 8 Istituto Cantonale di Microbiologia, Bellinzona CH-6501, Switzerland; 9 Blood Transfusion Service, Swiss Red Cross Berne, Berne CH-3001, Switzerland; 10 Laboratory of Virology, Geneva University Hospitals, Genève 14 CH-1211, Switzerland; 11 Institut Dr. Viollier AG, Basel CH-4002, Switzerland; 12 Center of Laboratory Medicine, St. Gallen, Switzerland; 13 Labor Dr. Güntert, Lucerne CH-6002, Switzerland; 14 Swiss HIV Cohort Study (SHCS) Data Center, University Hospital, Lausanne CH-1011, Switzerland; 15 Division of Infectious Diseases and and Hospital Epidemiology, University Hospital Zurich, University of Zurich, Zurich CH-8091, Switzerland

## Abstract

**Background:**

Serologic testing algorithms for recent HIV seroconversion (STARHS) provide important information for HIV surveillance. We have previously demonstrated that a patient's antibody reaction pattern in a confirmatory line immunoassay (INNO-LIA™ HIV I/II Score) provides information on the duration of infection, which is unaffected by clinical, immunological and viral variables. In this report we have set out to determine the diagnostic performance of Inno-Lia algorithms for identifying incident infections in patients with known duration of infection and evaluated the algorithms in annual cohorts of HIV notifications.

**Methods:**

Diagnostic sensitivity was determined in 527 treatment-naive patients infected for up to 12 months. Specificity was determined in 740 patients infected for longer than 12 months. Plasma was tested by Inno-Lia and classified as either incident (< = 12 m) or older infection by 26 different algorithms. Incident infection rates (IIR) were calculated based on diagnostic sensitivity and specificity of each algorithm and the rule that the total of incident results is the sum of true-incident and false-incident results, which can be calculated by means of the pre-determined sensitivity and specificity.

**Results:**

The 10 best algorithms had a mean raw sensitivity of 59.4% and a mean specificity of 95.1%. Adjustment for overrepresentation of patients in the first quarter year of infection further reduced the sensitivity. In the preferred model, the mean adjusted sensitivity was 37.4%. Application of the 10 best algorithms to four annual cohorts of HIV-1 notifications totalling 2'595 patients yielded a mean IIR of 0.35 in 2005/6 (baseline) and of 0.45, 0.42 and 0.35 in 2008, 2009 and 2010, respectively. The increase between baseline and 2008 and the ensuing decreases were highly significant. Other adjustment models yielded different absolute IIR, although the relative changes between the cohorts were identical for all models.

**Conclusions:**

The method can be used for comparing IIR in annual cohorts of HIV notifications. The use of several different algorithms in combination, each with its own sensitivity and specificity to detect incident infection, is advisable as this reduces the impact of individual imperfections stemming primarily from relatively low sensitivities and sampling bias.

## Background

Information on the incidence of HIV infection is crucial for monitoring the dynamics of the HIV epidemic in affected countries. Consequently, serologic testing algorithms for recent HIV seroconversion (STARHS) have been developed [1-4]. These tests make use of the fact that the HIV antibody response evolves during the first few months of infection with respect to concentration [5-7], proportion of the amount of total IgG [8], isotype [9], and avidity [10]. The time during which these properties remain below a predetermined cutoff may greatly differ individually, and its mean duration or 'window-period' has to be established by testing specimens from individuals with known date of HIV seroconversion [11]. Estimation of the incidence in a population is based on the relationship 'Prevalence = Incidence × Duration', as described by others [4,12]. The performance of STARHS, i.e. the sensitivity and specificity with which they recognize or exclude an incident infection in an individual patient is low and does not meet the standards required for tests used for diagnostic purposes. Therefore, STARHS should not be used for individual diagnosis. Recently, new procedures based on HIV genetic diversity, as detected by single-genome analysis, have been developed, which in the future may lead to more reliable results also enabling diagnosis of incident infection in individual patients [13-15].

STARHS require a special assay of reduced analytical sensitivity; hence they are also called 'detuned' assays. The reduced sensitivity renders these tests unsuitable for diagnosis of HIV infection and restricts their use to epidemiological studies. However, for systematic epidemiologic monitoring it would be convenient if information on incident infections could be gained prospectively and systematically from the same tests used anyway to diagnose HIV.

We have previously shown that a patient's antibody reaction in a widely used commercial line immunoassay, the Inno-Lia™ HIV I/II Score (Inno-Lia), provides information on the duration of infection similar to that of a commercial enzyme immunoassay (EIA), the so-called BED Incidence EIA [8,16]. The Inno-Lia is a type of second-generation Western blot (WB) that measures antibodies to different HIV antigens in a semi-quantitative way and is used for confirming HIV infection and to differentiate between HIV-1 and HIV-2 [17,18]. Timely diagnosis of HIV-2 is important, because this virus requires different tests for viral load quantification than the widely used and FDA-approved tests from Roche, Abbott, BioMérieux, or Bayer. Furthermore, HIV-2 treatment requires different antiretroviral drug regimens, as the virus is naturally resistant to some frequently used drugs including the whole class of non-nucleoside reverse transcriptase inhibitors (NNRTI) [19-22]. In some countries, the Inno-Lia is thus used routinely at the time of diagnosis, and in Switzerland the test has become a mandatory confirmatory test for HIV in 2006 [23].

As the pattern and intensity of HIV-specific antibodies both evolve during the first weeks to months after infection, it is possible to define algorithms which differentiate between early and late antibody patterns. Each of these algorithms has its own characteristic sensitivity and specificity for detecting incident infections. Of note, the utilization of the Inno-Lia results for population-based studies of HIV-1 incidence comes at no additional costs -- no additional test is needed.

As for STARHS, it is possible to determine window periods for the different Inno-Lia antibody patterns seen in early infection and to estimate the incidence based on these windows. Work in this direction is in progress. Alternatively, if the diagnostic sensitivity and specificity of an algorithm are known, which requires prior testing of suitable reference groups of infections of either less or more than 12 months duration, it is also possible to estimate the incidence by means of the basic diagnostic rule n_tested incident _= n_true incident _+ n_false incident_, whereby true-incident and false-incident are calculated based on the pre-determined values for diagnostic sensitivity and specificity [16]. The advantage of this approach is that the imperfections of most diagnostic tests -- a sensitivity and a specificity below 100% -- are already accounted for. In contrast, with other STARHS and particularly the widely used BED Incidence EIA [8], the incidence estimates based on the relationship 'Prevalence = Incidence × Duration' are frequently too high [24-29]. Correction factors for imperfect sensitivity and specificity in both early, i.e. patients who remain in recent state well beyond the window period, and late stage infection, i.e., patients in very advanced disease who return to recent state, have had to be implemented [30-35]. This has required determination of the locally measured false-negative and false-positive ratios, i.e. investigations of the same type as those that are the basis of our true-incident/false-incident approach.

A unique advantage of the Inno-Lia method is the fact that it tests the antibody response to various HIV antigens at the same time in a semi-quantitative way and therefore allows identification of various antibody patterns that are characteristic of early infection. Antibodies to five different HIV-1 antigens are assessed in the test, allowing many combinations characteristic of early stage infection to be defined. Thus, a number of different algorithms, each with its own sensitivity and specificity and yielding its own set of samples ruled recent can be applied to a test population, consequently reducing the sampling error associated with a single test. As the Inno-Lia is a confirmatory HIV test, it permits prospective testing of all newly diagnosed patients and notification of the results to the respective health authority, which may then periodically calculate the proportion of recent infections among the notified new cases or determine the incidence if the total number of HIV tests performed is also known.

Precise information on the diagnostic sensitivity and specificity of each algorithm is crucial for the method. If these parameters are not correct, estimates of incident infections will not be accurate. Originally, we estimated these parameters in a study of newly diagnosed patients with HIV-1 infection of either less or more than 12 months duration, as judged by the treating physicians of these patients. As the study was prospective and no follow-up data was available, it was uncertain whether these judgments were correct. In this regard, the diagnostic performance figures thus generated were of a preliminary nature, and the true diagnostic sensitivity and specificity of the algorithms remain to be established. Furthermore, as the specificity of STARHS might be impaired when testing patients infected with non-B subtypes of HIV-1 [3] or in advanced disease, it was deemed necessary to investigate whether or not these and other variables affect the outcome of the algorithms.

We have conducted two studies aimed towards establishing this goal. One study, published elsewhere [36], investigated the specificity of Inno-Lia algorithms in 714 patients of the Swiss HIV Cohort Study (SHCS) infected for at least 12 months and representing all clinical stages and major clades of HIV-1. That study showed that none of these parameters affected the algorithms. Although a viral RNA load below 50 copies/mL significantly reduced the specificity among patients receiving ART, age was the sole factor which could weakly impair the test specificity in untreated patients.

The second study, presented here, now addresses the diagnostic sensitivity of the Inno-Lia algorithms in a new cohort of patients infected for less than one year. It also investigates the overall diagnostic performance of the algorithms, i.e., their ability to distinguish between incident and older infection, and thus provides a basis for selecting the best algorithms for assessing annual cohorts of HIV notifications.

## Methods

### Ethics statement

The present study investigated patients of the Zurich Primary HIV Infection (ZPHI) study [37,38], the Swiss HIV Cohort Study (SHCS)[39] and data from anonymized HIV notifications to the Swiss Federal Office of Public Health (SFOPH. The two studies were approved by the respective ethical committees involved -- the ZPHI by the ethical committee of the Zurich University Hospital and the SHCS by the ethical committees of all participating institutions (see http://www.shcs.ch). All participating patients gave their written informed consent to the respective study goals, which also include the present nested study. No informed consent was required for the anonymized notifications.

### Patients with incident infection

Patients with HIV-1 infection of ≤ 12.0 months duration (= recent or incident infection) were used for determination of the diagnostic sensitivity of the Inno-Lia algorithms. These patients originated from either the ZPHI study or the anonymized HIV notifications to the SFOPH from April 2007 to December 2010.

The ZPHI study is an observational, open label, nonrandomized, single-center study (ClinicalTrials.gov identification no. NCT00537966) [40]. Patients with acute or recent HIV-1 infection were included. Acute HIV-1 infection was defined as 1) presentation of the acute retroviral syndrome (ARS) and a negative or indeterminate WB or Inno-Lia results in the presence of a positive p24 antigen test and/or a detectable viral load; or 2) a documented seroconversion with or without symptoms no more than 90 days ago. Recent infection was defined as 3) a possible ARS, a positive WB or Inno-Lia result, detectable viral load, and a positive HIV gp120 avidity respectively detuned assay result [41]; or 4) a documented acute HIV-1 infection with referral to our center within 90 days after estimated date of infection (EDI). For each patient, EDI was determined by taking into account the pattern of different assay results (first positive and last negative HIV-test; negative, indeterminate and positive WB; positive p24 Ag; antibody avidity assay), patient's reports of unambiguous risk contacts, and timing of onset of ARS symptoms. With respect to WB results, the following rules were applied to determine the EDI: (i) Negative WB (Fiebig stages I-III) [42]: If a single risk contact was reported within the last three weeks before the date of WB, this date was taken as EDI. In contrast, if no history of risk contacts was reported, infection was assumed to have occurred 14 days before the WB date. (ii) Indeterminate WB (Fiebig stage IV): If a single risk contact was reported between 2 and 6 weeks before the date of WB, this date was taken as EDI. In case of several risk contacts, a higher and lower range was estimated and the mean of this range was taken as EDI. (iii) Positive WB (Fiebig stages V-VI): If a single risk contact occurred 6 weeks or earlier before the date of the WB, this date was taken as EDI if seroconversion was documented. If a seroconversion within 6 months was clearly documented without history of risk contact, the mean date between the two tests (last negative and first positive HIV-test) was taken as EDI. If a patient had a history of an ARS, a fully converted WB, but no documented seroconversion and a negative detuned or avidity assay, the EDI was defined as the date 20 days before the onset of the ARS. These EDI definitions have been successfully used and validated in previous publications [14,38,40,43].

Incident infection among the HIV notifications was identified as a case that met one of the following definitions. (1) Laboratory evidence of seroconversion at the time of diagnosis, i.e., a reactive 4^th^-generation HIV-1/2/O antibody/p24 antigen combination screening test and a positive virus component test (HIV-1 RNA or DNA or p24 antigen) in combination with a negative 3^rd^-generation HIV-1/2/O antibody-only enzyme immunoassay and/or a negative or indeterminate Inno-Lia result according to the manufacturer's instructions for result interpretation. (2) A self-reported or documented negative HIV screening result no more than 12 months before diagnosis. (3) Documented signs of ARS no more than 90 days before diagnosis [44]. EDI among the notifications was defined as 14 days before the reported date of onset of PHI symptoms or the mean date between the last negative and first positive HIV-test.

### Patients with older infection

Patients with HIV-1 infection of > 12.0 months duration (= older infection) were needed for determination of the diagnostic specificity of the algorithms. They were selected from among either the SHCS or the HIV notifications.

The SHCS patients, already investigated in a previous study [36], had been infected with HIV-1 for at least 12 months, as shown by either a documented first positive HIV test or registration into the SHCS at least 12 months prior to sample date. The patients represented all major clades of HIV-1 as well as the clinical stages and CD4+ strata. Virus concentrations were ≥50 HIV-1 RNA copies/mL and thus sufficiently high not to lead to false-recent antibody patterns [36]. Patients selected from the HIV notifications met at least one of the following definitions: (1) Presence of signs and symptoms of CDC stage B or C or (2) progression to CDC stage C in the year of HIV diagnosis or the year thereafter.

### Patients with unknown duration of infection

The majority of the SFOPH notifications consisted of patients who did not meet the above-mentioned definitions for incident or older infection. These cases of unknown duration of HIV infection were utilized mainly for the evaluation of the Inno-Lia algorithms in annual notification cohorts. However, some of these notifications also contained some kind of information on the duration of infection, such as a negative HIV test earlier than 12 months before diagnosis or onset of presumed PHI at 4 to 12 months before diagnosis. EDI for these cases was determined in the same way as for the notifications classified as incident infections.

### Inno-Lia testing

ZPHI and SHCS samples were number-coded and tested batch-wise by the Inno-Lia HIV I/II Score assay (Innogenetics, Ghent, Belgium). If there was more than one sample per patient, as was the case in the ZPHI study, the latest available sample before onset of antiretroviral treatment was selected. All retrospective Inno-Lia testing was performed between October 2008 and January 2009. Notification samples were tested prospectively at the time of diagnosis. All testing -- prospective or retrospective -- was conducted in 11 HIV confirmatory labs commissioned by the SFOPH or in the Swiss National Center for Retroviruses (SNCR), which serves as the national HIV reference laboratory and is also commissioned by the SFOPH. All these laboratories are accredited according to the ISO/IEC 17025 standard by the governmental Swiss Accreditation Service SAS http://www.seco.admin.ch/sas/index.html?lang=en. All labs had participated in previous collaborative studies of Inno-Lia based recent infection assessment and were experienced with the test [16,36].

All Inno-Lia testing was performed according to the manufacturer's 16-h sample incubation protocol. The Inno-Lia is a WB-like line immunoassay that measures antibodies against recombinant proteins or synthetic peptides of HIV-1 group M, HIV-1 group O, or HIV-2, which are coated as 7 discrete lines on a nylon strip. As each test strip also contains three quantitative internal standards, a semi-quantitative ranking of the different antibody reactions is possible [17,18]. Antibody reactions relevant for assessment of recent infection status include those to sgp120, gp41, p31, p24 and p17.

### Collection of Inno-Lia results by means of HIV notifications

Since September 2007, all newly diagnosed HIV patients were notified to the SFOPH by means of a Microsoft Excel^® ^based electronic form. Anonymized personal identification and all available diagnostic data of each patient including the detailed Inno-Lia results were entered into this form by the 11 HIV notification labs or the SNCR and forwarded by e-mail to the SFOPH. At the SFOPH, these data were transferred into a database subsequently linked with socio-epidemiological data from notifications received from the treating physicians of the newly diagnosed patients and used for evaluation by the different incident infection algorithms.

### Incident infection algorithms

A total of 26 algorithms (Algs) for incident HIV-1 infection were employed. They had been developed by investigating which Inno-Lia antibody patterns were found at maximal frequency in patients with up to 12 months duration of infection and at minimal frequency in patients with longer than 12 months of infection. Of the total, 24 algorithms were as described [16,36], whereas Algs 11.2 and 15.1 were developed anew.

### Calculation of the incident infection rate (IIR)

Calculation of the proportion of incident infections in a population testing positive for HIV-1, the incident infection rate (IIR), was based on the relationship n_tested incident _= n_true- incident _+ n_false- incident_, wherein n_true- incident _= n_tested _× IIR × %Sensitivity/100 and n_false- incident _= n_tested _× (1 - IIR) × (1 - %Specificity/100). Therefore, equation #1 is: IIR = (n_tested incident_/n_tested _+ %Specificity/100 - 1)/(%Sensitivity/100 + %Specificity/100 - 1) [16].

For calculating the IIR by means of the window-based BED Incidence EIA, equation #2, as explained in detail elsewhere [1,45] was employed: IIR = n_tested incident_/n_tested _× 365/window; using a window of 153 days, as instructed by the test's manufacturer, Calypte Biomedical Corporation, Lake Oswego, Oregon, USA.

### Data evaluation and statistics

Inno-Lia results and clinical or socio-epidemiological data were linked only after all testing was completed. Specificity, sensitivity, total of correct results achieved with the various incident infection algorithms and logistic likelihood ratio (LLR) chi-square values were calculated by means of contingency tables and logistic regression, as contained in the StatView^® ^5.0 program for Macintosh (SAS Institute Inc., Cary, North Carolina, U.S.A.). StatView was also used to determine the result (incident or older) of a sample by each of the 264 Inno-Lia algorithms. Means were compared by paired or unpaired t-tests, as specified under Results, and correlation was assessed by Pearson's test using Fisher's r to z transformation.

## Results

Plasma or serum samples from a total of 2'641 HIV-1 infected patients were tested by the Inno-Lia HIV I/II Score assay, and the resulting antibody patterns were evaluated by 26 previously described recent infection algorithms [36]. Out of the total, 527 patients (20.0%) met the criteria for incident infection of up to 12 months duration (see Methods), 740 (28.0%) those for older infection, and the remaining 1'374 patients (52.0%) had an infection of unknown duration. The main characteristics of the three patient groups are summarized in Table 1.

**Table 1:** Overview of patient characteristics

A) Patients with incident infection (n = 527)				
	**ZPHI patients**		**HIV notifications**	

Number	144		383	

Male (n, %)	129	(89.6)	332	(86.7)
Female (n, %)	15	(10.4)	51	(13.3)

Transmission risk (n, %)				
MSM	102	(70.8)	242	(63.2)
HET	37	(25.7)	102	(26.6)
IVDU	4	(2.8)	17	(4.4)
OTH	1	(0.7)	0	(0)
Unknown	0	(0)	22	(5.7)

Age (median, IQR)	36	(30 - 42)	35	(28 - 43)

Months of infection (median, IQR)	1.59	(1.05 - 2.73)	1.04	(0.33 - 3.0)

HIV-1 RNA, (log [copies/mL]), IQR	5.1	(4.5 - 5.8)	5.2	(4.5 - 6.1)

CD4+ T cell count, cells/μL (median, IQR)	372	(270 - 552)	n.a.	

**B) Patients with older infection (n = 740)**				

	SHCS patients*		**HIV notifications**	

Number	412		328	

Male (n, %)	205	(49.8)	231	(70.4)
Female (n, %)	207	(50.2)	97	(29.6)

Transmission risk (n, %)				
MSM	54	(13.1)	90	(27.4)
HET	283	(68.7)	194	(59.1)
IVDU	48	(11.6)	16	(4.9)
OTH	24	(6.6)	6	(1.8)
Unknown	0	(0)	21	(6.4)

Age (median, IQR)	35	(30 - 41)	44	(34 - 53)

Days of infection (median, IQR)	n.a.	n.a.	n.a.	n.a.

HIV-1 RNA, (log [copies/mL]), IQR	3.9	(2.9 - 4.7)	5.16	(4.8 - 5.6)

CD4+ T cell count, cells/μL (median, IQR)	350	(208 - 544)	n.a.	

Treatment status				

Prior to ART	190	(46.1)	328	(100)

Under ART	222	(53.9)	0	(0)

**C) Notifications with unknown duration of infection (n = 1374)**				

Number			1374	

Male (n, %)			924	(67.2)
Female (n, %)			423	(30.8)
Unknown			27	(2.0)

Transmission risk (n, %)				
MSM			401	(29.2)
HET			472	(34.3)
IVDU			40	(2.9)
OTH			8	(0.6)
Unknown			453	(33.0)

Age (median, IQR)			36	(30 - 45)

HIV-1 RNA, (log [copies/mL]), IQR			4.8	(4.1 - 5.4)

n.a., not available; *same patients as investigated in [36]

### Diagnostic sensitivity and specificity of the algorithms for detecting incident infection

For determining the diagnostic sensitivity of the algorithms, 527 samples obtained from treatment-naïve patients with up to 12 months duration of HIV-1 infection were available (Table 1, section A). These incident infection specimens included 144 from the ZPHI study and 383 from the HIV notifications (see Methods). The two groups were similar with respect to the distributions of sex, transmission risk, age, and concentration of HIV-1 RNA. Regarding duration of infection, the incident HIV notification cases on average represented an earlier window of the infection than the ZPHI samples (p < 0.001; unpaired *t*-test).

In order to determine the diagnostic specificity of the algorithms, 740 samples from patients with infection longer than 12 months were used (Table 1, section B). Out of this total, 412 samples originated from the previously published SHCS study [36] and 328 samples originated from the HIV notifications (see Methods). The two groups differed with respect to the distributions of sex, transmission risk, age, and HIV-1 RNA load (unpaired *t*-test; p < 0.01 for all). Furthermore, a majority of the SHCS patients were receiving ART (which explains their lower mean viral load), while all notification cases were ART-naïve. All SHCS patients included in the analysis had a viral load of 50 copies/mL or higher, because concentrations below this limit were excluded due to their association with false-recent results [36].

The diagnostic sensitivity of each of the 26 Inno-Lia algorithms among the 527 incident infection samples is depicted in Table 2. Also shown are the diagnostic specificity among the 740 older infection samples, the percentage of overall correct results, the logistic likelihood ratio (LLR) chi-square value as a further measure of the diagnostic performance, and the final rank of each algorithm. The sensitivity of the algorithms extended from 20.3% for Alg3 to 63.95% for Alg4.1, while the specificity was between a minimum of 90.80% for Alg6 and a maximum of 100% for Algs 3 and 3.1. The newly developed Alg15.1 distinguished best between incident and older infection. It exhibited a sensitivity of 61.67%, a specificity of 95.14%, and it correctly classified 81.22% of the 1'267 samples (rank 1). The respective LLR Chi-square value was 524.69 (rank 2), and the sum of the two ranks was 3, thus placing Alg15.1 at the top of all algorithms. A number of other algorithms including Algs 15, 11.2, 13, 7, 9, 11.1, 12.1, 4.1, 12, 8.1 and 11 distinguished similarly well between incident and older infection. As Algs 11 and 11.1 on the one hand and also Algs 12 and 12.1 on the other hand were very similar to each other, Algs 11 and 12 were further excluded, leaving 10 best Algs for use in further analyses. Depending on whether the ranking was based on overall correct results or LLR Chi-square, different though overlapping sets of best algorithms would have been selected. It was thus justified to use both criteria in combination.

**Table 2:** Diagnostic performance of Inno-Lia algorithms for recent HIV-1 infection among the 1267 patients with incident or older infection of Table 1

Alg #	Definition	Sensitivity %	Specificity %	Overall correct % (rank)	LLR Chi-square (rank)	Rank sum (final rank)
15.1	if (sgp120 ≤ 1 AND p31 ≤ 1 AND p17 ≤ p24) OR (gp41 ≤ 2 AND p31 ≤ 1 AND p17 ≤ p24) OR (p17 ≥ 2 AND p31 = 0 AND p17 ≤ p24) OR (p31 = 0 AND p24 ≥ 2 AND p17 ≤ p24) then RECENT else older	61.67	95.14	81.22 (1)	524.69 (2)	3 (1)

15	if (sgp120 ≤ 1 AND p31 ≤ 1) OR (gp41 ≤ 2 AND p31 ≤ 1) OR (p17 ≥ 2 AND p31 = 0) OR (p31 = 0 AND p24 ≥ 2) then RECENT else older	62.24	94.32	80.98 (2)	509.19 (4)	6 (2)

11.2	if (sgp120+gp41 ≤ 2.5) OR ((sgp120+gp41+p31+p24+p17 ≤ 6.5) AND p31 ≤ 1 AND p17 ≤ p24) OR (p31 = 0 AND p24 ≥ 2) then RECENT else older	61.48	94.05	80.51 (3)	490.62 (6)	9 (3.5)

13	if (sgp120+gp41 ≤ 4 AND p31 = 0) OR (p31 = 0 AND p24 ≥ 2) then RECENT else older	59.77	95.00	80.35 (4)	493.18 (5)	9 (3.5)

7	if sgp120+gp41+p31 ≤ 4 then RECENT else older	54.08	98.38	79.95 (10.5)	530.11 (1)	11.5 (5)

9	if sgp120+gp41 ≤ 4 AND p31 = 0 then RECENT else older	52.75	98.38	79.40 (12)	511.40 (3)	15 (6)

11.1	if (sgp120+gp41 ≤ 2.5) OR ((sgp120+gp41+p31+p24+p17 ≤ 6.5) AND p31 ≤ 1 OR (p31 = 0 AND p24 ≥ 2) then RECENT else older	61.48	93.51	80.19 (6.5)	475.99 (9)	15.5 (7)

12.1	if (p24 ≥ 2 AND p31 = 0) OR (gp41 ≤ .5) OR (sgp120+gp41+p31 ≤ 4) OR (p31 ≤ 1 AND (sgp120+gp41+p31+p24+p17 ≤ 6.5)) then RECENT else older	61.67	93.38	80.19 (6.5)	475.12 (10)	16.5 (8)

4.1	if p31 ≤ 0.5 then RECENT else older	63.95	91.89	80.27 (5)	470.01 (14)	19 (9.5)

12	if (p24 ≥ 2 AND p31 = 0) OR (gp41 ≤ .5) OR (sgp120+gp41+p31 ≤ 4 OR sgp120+gp41+p31+p24+p17 ≤ 6.5) then RECENT else older	61.67	93.37	80.17 (8)	474.55 (11)	19 (9.5)

8.1	if gp41 ≤ 0.5 OR (sgp120+gp41+p31 ≤ 4) OR ((sgp120+gp41+p31+p24+p17 ≤ 6.5) AND p31 ≤ 1) then RECENT else older	54.65	96.76	79.24 (13)	476.63 (7)	20 (11)

11	if (sgp120+gp41 ≤ 2.5) OR (sgp120+gp41+p31+p24+p17 ≤ 6.5) OR (p31 = 0 AND p24 ≥ 2) then RECENT else older	61.48	93.37	80.09 (9)	471.87 (12)	21 (12)

8	if gp41 ≤ 0.5 OR (sgp120+gp41+p31 ≤ 4) OR (sgp120+gp41+p31+p24+p17 ≤ 6.5) then RECENT else older	54.65	96.75	79.23 (14)	476.13 (8)	22 (13)

13.1	if gp41 ≤ 2 OR (p31 = 0 AND p24 ≥ 2) then recent else older	60.34	93.92	79.95 (10.5)	470.77 (13)	23.5 (14)

3	if gp41 ≤ 0.5 then RECENT else older	20.30	100.0	66.85 (25)	201.79 (23)	24.5 (22)

4	if p31 = 0 then RECENT else older	60.15	92.70	79.16 (15)	436.83 (16)	31 (15)

17	if (sgp120 * gp41) ≤ 2 then RECENT else older	50.09	98.24	78.22 (17)	469.33 (15)	32 (16)

2	if sgp120 ≤ 1 then RECENT else older	54.46	95.41	78.37 (16)	431.97 (17)	33 (17)

16	if (sgp120 ≤ 1 AND (p31+p24+p17 ≤ 2.5)) OR (gp41 ≤ 1) OR (p31 ≤ 0.5 AND (sgp120+gp41+p24+p17 ≥ 15)) OR (p24 = 0 AND gp41 ≤ 2) OR (p24 ≥ 3 AND p31 = 0) then RECENT else older	50.47	96.62	77.43 (18)	416.47 (19)	37 (18.5)

18	if (sgp120 * gp41 ≤ 1) OR (p24+p31 = 0) then RECENT else older	46.11	98.24	76.56 (19)	416.96 (18)	37 (18.5)

14	if (sgp120+gp41+p31+p24+p17 ≤ 6.5 AND p31 ≤ 1) then RECENT else older	45.92	97.84	76.24 (20)	399.26 (20)	40 (20)

3.2	if gp41 ≤ 2 then RECENT else older	44.02	98.11	75.61 (21)	385.38 (21)	42 (21)

6	if p17 = 0 then RECENT else older	40.80	90.80	69.98 (22)	178.75 (24)	46 (22.5)

3.1	if gp41 ≤ 1 then RECENT else older	23.53	100.0	68.19 (24)	236.77 (22)	46 (22.5)

10	if p31 = 0 AND p24 ≥ 2 then RECENT else older	30.93	95.27	68.51 (23)	164.49 (25)	48 (24.5)

5	if p24 ≤ 0 then RECENT else older	26.00	95.54	66.61 (26)	125.29 (26)	52 (26)

### Adjustments for overrepresentation of very early incident infections

The months of infection information in Table 1 indicates that the incident infection samples were not distributed evenly across the entire 12-months period of incident infection, but concentrated within the first few months. In order to assess the effect of the duration since EDI, we separately analyzed the diagnostic sensitivity for each quarter, using the selected 10 best algorithms on a total of 621 patients for whom an EDI within the 12 months preceding the diagnosis was known (Table 3). The analysis included the 144 ZPHI patients and 477 notifications, including 94 samples with a less precise EDI from section C of Table 1. The number of cases in each quarter is shown in the header of the table. The diagnostic sensitivity was relatively high in the first quarter, achieving a mean of 71.3% (range 63.6 to 75.9%), but dropped to means below 20% in the second, third and fourth quarters. The true sensitivity in the third and fourth quarter was most likely even lower, as all of the 22 notification samples ruled incident by at least one algorithm were placed into these quarters because of a negative HIV test 12 to 24 months before diagnosis, thus yielding a midpoint of 6 to 12 months before diagnosis. In contrast, none of the better characterized 15 ZPHI patients placed within these two quarters was ruled recently infected (2 × 2 table test; p < 0.05).

**Table 3:** Diagnostic sensitivity of the 10 best algorithms per quarter of incident infection

Alg #	**Non-adjusted sensitivity S**_**0**_	Quarter 1 n = 406	Quarter 2 n = 112	Quarter 3 n = 58	Quarter 4 n = 45	**Adjusted sensitivity S**_**1**_	**Adjusted sensitivity S**_**2**_	**Adjusted sensitivity S**_**3**_
4.1	63.95	75.86	22.30	22.40	17.80	34.6	55.1	42.1

15	62.24	74.88	20.50	17.20	17.80	32.6	53.6	40.2

15.1	61.67	74.14	20.50	17.20	15.60	31.9	52.9	39.6

12.1	61.67	74.14	20.50	15.50	15.60	31.4	52.8	39.3

11.2	61.48	73.89	20.50	15.50	15.60	31.4	52.6	39.2

11.1	61.48	73.89	20.50	15.50	15.60	31.4	52.6	39.2

13	59.77	71.92	18.80	13.80	15.60	30.0	50.9	37.7

8.1	54.65	65.76	17.90	10.30	6.70	25.2	45.8	33.1

7	54.08	65.27	16.10	10.30	6.70	24.6	45.2	32.4

9	52.75	63.55	16.10	8.60	6.70	23.7	43.9	31.4

**Mean**	**59.37**	**71.33**	**19.4**	**14.6**	**13.4**	**29.7**	**50.5**	**37.4**

Although the diagnostic sensitivity in these later quarters of the incident infection period can thus not be established accurately, it is clear that the raw sensitivities of Table 2 (shown again in column S_0 _of Table 3) do not apply to them, and a bias would be introduced if the effect of duration since EDI were not adjusted for. Thus, we developed several models for adjusting the raw sensitivities. In the first adjustment model, we obtained adjusted sensitivities S_1 _simply by averaging the four quarter sensitivities. This corresponds to a model that assumes an even distribution of diagnosing incident infections over all four quarters. The respective adjusted sensitivities S_1 _extended from a maximum of 34.6% for Alg4.1 to a minimum of 23.7% for Alg9. The mean S_1_, 29.7%, was roughly half the mean of S_0_. If the sensitivity in quarters 3 and 4 was set to zero, as suggested by the results of the 15 ZPHI patients, mean S_1 _was reduced to 22.7%.

Obviously, however, the samples used in the analysis are not distributed evenly among all four quarters and thus do not contribute equal amounts of information for estimating overall sensitivities, leading to a further potential bias. We therefore made further adjustments by weighting the contribution of each quarter to the overall sensitivity. The analysis was restricted to the HIV notifications, as these are representative with respect to the duration of the newly diagnosed infections. Weighting was based on the number of the notifications in each quarter. In our second adjustment model, we considered all three definitions for an incident infection (i.e., laboratory, clinical, and based on a negative test). In the third model, we excluded all cases judged incident because of reported signs or symptoms of ARS (incidence definition 3 for the HIV notifications in Methods) and considered only the notifications with a previous negative HIV test. Model 3 thus did not select for symptomatic acute infections.

With model 2, there were a total of 477 incident infections. Thirty-one of these were established based on lab results, 225 were based on information regarding onset of ARS, and 221 were based on a negative test within 24 months before diagnosis. Two-hundred ninety-five of the 477 cases (61.8%) were in the first quarter, 94 (19.7%) in the second, 50 (10.5%) in the third, and 38 (8.0%) in the fourth. The adjusted, weighted sensitivities S_2 _were calculated by multiplying the quarter sensitivities with these percentages and then averaging the products. They extended from a minimum of 43.9% to a maximum of 55.1%; the mean was 50.5%. If, again, the sensitivity in quarters 3 and 4 was set to zero, the mean S_2 _was reduced to 47.9%.

With model 3, there were a total of 315 samples. Of these, 120 (38.1%) were placed in the first quarter, 91 (28.9%) in the second, 57 (18.1%) in the third, and 47 (14.9%) in the fourth. When weighting the quarter sensitivities with the respective percentages, the sensitivities S_3 _were obtained. They extended from a minimum of 31.4% to a maximum of 42.1%, with a mean of 37.4%. A reduction to a mean of 32.8% was obtained when assuming a zero sensitivity for quarters 3 and 4.

In the context of calculating annual incident infection rates in a population of interest, each of these models has its merits and justifications, and we therefore used all of them in parallel.

### Application of algs for estimating the annual incident infection rates (IIR)

Using the adjusted sensitivities S_1_, S_2 _or S_3 _in combination with the specificities of Table 2 and employing equation #1 of Methods, we calculated the IIR for the 10 best-performing algorithms in four annual cohorts of newly diagnosed HIV-1 infections notified to the SFOPH (Additional file 1). Based on which sensitivity was used, the mean IIR at baseline (cohort A) varied between 0.246 and 0.453. For 2008 (cohort B), with each model, all 10 algorithms indicated an increase of IIR averaging 30.6%, which was highly significant (P < 0.0001; two-sided paired *t*-test). It should be considered, however, that some of the algorithms are similar and thus do not yield truly independent measurements. For 2009 (cohort C), and again independently of the sensitivity employed, six algorithms indicated a relative decrease, while three indicated a further, modest increase and one no change. Overall, this resulted in a mean IIR that amounted to 121.9% of the IIR in cohort A. Compared to 2008, the difference was weakly significant (P = 0.039; two-sided paired *t*-test). In 2010 (cohort D), all 10 algorithms again indicated a distinct decline to a mean IIR back at baseline (P < 0.0001; two-sided *t*-test). When sensitivities calculated on the assumption of a zero detection rate in quarters 3 and 4 were used, the average IIR of the four cohorts increased to 0.63, 0.82, 0.77 and 0.64 when using S_1_, to 0.26, 0.34, 0.32 and 0.26 when using S_2 _and to 0.40, 0.52, 0.49 and 0.41 when using S_3_. In all these instances, the relative differences between the four cohorts were identical as shown in Additional file 1. Thus, the relative changes between different annual cohorts were independent of the absolute value of the diagnostic sensitivity.

**Figure 1:**
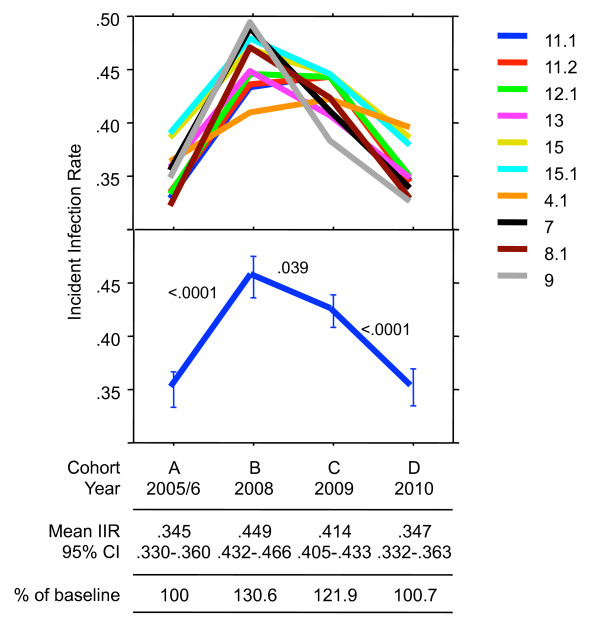
**Incident infection rates (IIR) in four annual cohorts of newly diagnosed HIV-1 infections based on adjusted sensitivity S_3_**. Individually shown in the top panel are the IIR of the 10 best algorithms, while the respective means with their 95% confidence intervals are shown in the panel below. The P-values in the lower panel refer to differences between the annual cohorts, as determined by paired *t*-test.

Although the 10 algorithms performed similarly -- all exhibited an increase between the baseline cohort and 2008 and a decline between 2009 and 2010, they differed to some degree with respect to the IIRs of the four cohorts and even more with regard to the resulting differences between them. This is illustrated in Figure 1, which is based on the sensitivities S_3_; for details refer to section C of the Additional file 1. For instance, the highest relative increase in the IIR between baseline and 2008 was from 0.312 to 0.459 for Alg8.1 (+46.9%), while the smallest, for Alg4.1, was from 0.356 to 0.402 (+12.9%). Similarly, the individual IIRs for the period of declining average IIR varied considerably. With Alg7, there was a steep decline between 2008 and 2009, but with Algs 12.1, 4.1, 11.1 and 11.2 there was either no change or even a further increase. These differences illustrate that a combination of several different algorithms rather than a single one should be used for determining the IIR.

## Discussion

The purpose of the present study was to further assess the diagnostic performance of Inno-Lia based algorithms for incident HIV-1 infection and to see whether previous estimates for sensitivity and specificity would be confirmed in a new, much larger dataset of newly diagnosed patients with no overlap to our first study [16]. In a recent study of patients known to have been infected for at least one year, we have demonstrated a high diagnostic specificity of the algorithms, which was not affected by the HIV-1 clade or the disease stage [36]. The present work also confirms the high specificity of the algorithms (Table 2). Although the specificities presented here are on average slightly lower than those found previously (mean 95.74 vs. 96.47; P < 0.0001; paired *t*-test), they are of a similar magnitude, and there is an excellent correlation across algorithms between the earlier reported and new values (R = 0.983; P < 0.0001). As there is no follow-up among the notification cases, the lower specificity in the present investigation may be due to patients with severe acute retroviral syndrome, who were erroneously classified as CDC stages B or C [46-48]. The small reduction has little impact on the performance of the algorithms.

Regarding the diagnostic sensitivity, the raw sensitivities in Table 2 are clearly overestimates of the sensitivities that would apply to a population of patients distributed evenly over their first year of infection. The overestimation is due to an overrepresentation of patients in the first three months of infection, as is indicated by the numbers of available samples shown in the header of Table 3. The most important reason for this is that patients with symptomatic ARS are preferentially diagnosed because they will seek medical attention. Similarly, in case of a known exposure to HIV, patients will seek to clarify their HIV status as rapidly as possible, usually within 3 months, thereby again favoring detection of their infection in the first quarter. The only way to obtain reliable sensitivity rates for the later quarters of the incident infection period is by follow-up of patients diagnosed early and by subsequent repeated testing during the entire first year of infection. This was sought with the study of the ZPHI patients, but because most of these patients opted for early ART, the number of suitable samples representing the later quarters was small and could not provide reliable results. This forced us to rely predominantly on notification cases. For these, the reliability of information on the EDI decreases with time since the last negative HIV test or onset of ARS. Thus, the sensitivity in quarters 3 and 4, and thus the overall diagnostic sensitivity of the algorithms, can currently not be determined exactly, and Tables 3 and Additional file 1 illustrate the breadth of uncertainty that persists at this stage.

The present investigation shows that the algorithms preferentially detect incident infections in their first quarter. Here, the sensitivity was good, while in the second, third, and fourth quarters it was low, as explained above. If patients diagnosed in their first year of HIV infection were distributed evenly across all four quarters, an adjustment with equal weight for all quarter sensitivities, as is represented by sensitivity S_1_, would be appropriate. However, the clear overrepresentation of samples in the first quarter suggests that diagnosis of incident HIV infection is biased towards the first few months. It is therefore advisable to weight the quarter sensitivities with the proportion of patients diagnosed in the respective quarter. We believe that adjustment model 3, which yields the sensitivities S_3_, is most appropriate, as it is based on a definition of incident infection that is not affected by symptoms of HIV disease. Therefore, the later quarters are better represented than with model 2, while with model 1 they would probably be overrepresented.

Using S_3_, our estimate of the incident infection rate in the baseline cohort A of HIV notifications to the SFOPH was remarkably close to a previous estimate, although that earlier estimate was based on a different adjustment method, which involved different sensitivities for early, intermediate and late stages of incident infection [16]. In that earlier approach we used Algs 9 and 12 and estimated the IIR in cohort A at 0.33 and, respectively, 0.35. In the present study, the IIR for these two algorithms were 0.34 and 0.36, respectively, while the mean IIR based on the 10 best algorithms was also 0.35 (see Additional file 1). In view of the different approaches, i.e., one with reactivity-based and the other with time-based corrections, the agreement of the two IIR estimates is remarkable.

### Advantages of using a combination of algorithms

One might argue that the simplest way of estimating the IIR would be by selecting the best algorithm and using only that best algorithm for all IIR calculations. However, a closer inspection of Table 2 shows that it is difficult to select a single best algorithm among the group of top-performing algorithms. One also has to consider that the best-performing algorithm in one population need not necessarily be the best in a different one: Apart from diagnostic sensitivity and specificity, the proportion of overall correct results depends highly on the true proportion of incident infections in a test population. If that proportion is low, a test with maximal specificity will prevail over one with maximal sensitivity. Conversely, at a high proportion, a test with maximal sensitivity will outperform one optimized for specificity. Thus, these algorithms will work optimally when used in combination. Figure 1 perfectly illustrates this point by clearly demonstrating that it would be impossible to select a best curve among the 10 shown. It should also be considered, however, that the algorithms are not truly independent of each other, and this has to be kept in mind when performing statistical evaluations. Whether one uses a single algorithm or a combination of different ones has no effect on costs, as these population-based evaluations are done in an automated way, e.g. by entering the Inno-Lia dataset into a simple pre-formed Excel table.

### Comparison with the BED incidence EIA

Notification cohort A had originally also been tested by the BED-EIA, which had ruled 262 of the 748 samples as incident [16]. When using the BED-EIA in the sense of its developers, i.e., based on a window of 153 days and using equation #2 shown under Methods, an IIR of 0.84 was obtained. This was unacceptably high given the fact that symptoms of CDC stages B or C, i.e. distinct manifestations of older infection, had been reported in 21.5% of these notifications and that a sizable fraction of the 392 patients with no symptoms (52.4% of the total) must also have been in the stage of older infection [16]. A recent study conducted in Switzerland showed that 31% of newly HIV diagnosed individuals presented with less than 200 CD4+ T cells and 10% even with less than 50 CD4+ T cells per microliter [49]. We thus have to conclude that the BED-EIA derived, window-based IIR of 0.84 is grossly overestimated. This is in accordance with the findings of others, who also observed IIRs twice or three times too high [27].

Of note, when the BED-EIA results were evaluated in the same way as the Inno-Lia algorithms, i.e. based on the test's empirically determined sensitivity and specificity and employing equation #1, an IIR of 0.37 was calculated [8,16]. This was in excellent agreement with both our previously published and new Inno-Lia based estimates. The question thus arises whether it would not be simpler to use the BED assay in connection with IIR equation #1 instead of employing #2 and then having to correct for the imperfect sensitivity and specificity of the test in the investigated population [12,29-35,50].

### Changes in IIR over time

All 10 Inno-Lia algorithms indicated a distinct increase in IIR for the notifications of 2008 compared to 2005/2006 and a decrease back to baseline in 2010 (Figure 1). Additional file 1 demonstrates that while the absolute IIR differ considerably depending on the adjustment model for the sensitivity, the relative differences between the different annual cohorts are identical for all three models, as well as for their further modifications based on the assumption of a zero detection rate in quarters 3 and 4. Thus, when only interested in changes in IIR over time, an exact knowledge of the diagnostic sensitivity is not required. Nevertheless, in view of the excellent agreement of previous and actual IIR estimates in notification cohort A and the further good agreement with the BED-based IIR in this cohort when using equation 1, we clearly favor the sensitivities S_3_. When used in conjunction with the specificities of Table 2, they should permit a continuous assessment of IIR changes of different transmission risk groups over time in Switzerland. This should also permit a timely evaluation of the effectiveness of HIV prevention campaigns targeted at different risk groups.

As the assessment of relative changes is not dependent on the true sensitivity and because the Inno-Lia algorithms are unaffected by HIV-1 clade and other variables that affect the specificity of other STARHS [36], the method should also be usable in countries other than Switzerland. Caution is required, however, when using the method for determination of absolute incident infection rates, and further studies should be done.

### Conclusions and limitations

In conclusion, we have determined the diagnostic performance of 26 Inno-Lia algorithms for recent infection in patients with verified recent or older HIV-1 infection. Although the diagnostic sensitivity of some algorithms determined here may lack ultimate precision despite our correction for overrepresentation of 1st quarter samples, the use of the 10 best-performing algorithms in combination permits minimization of the impact of their individual limitations and the effects of sample bias and to monitor changes in IIR in a population over time. As precise knowledge of the diagnostic sensitivity is not required for the assessment of such relative changes, the specificity and adjusted sensitivity figures established here should also be usable for similar studies in other regions of the world. Our method is particularly attractive for countries in which the Inno-Lia is already used as a confirmatory assay and for differentiation between HIV-1 and HIV-2.

As our method is based on a distribution of early and late infections that is characteristic of newly diagnosed HIV-1 infections in a population including all ages of adulthood, our method is not applicable to selected age groups, e.g., young adults newly exposed to HIV risk encounters. As explained above, the method should not be used for determining absolute incident infection rates. Finally, as the diagnostic performance of the method, particularly the sensitivity of the algorithms, does not meet the standards for tests used for diagnostic purposes, the method is unsuitable for diagnosing incident or older infection in individual persons. The method should only be used in population-based studies.

## Abbreviations

STARHS: Serological Testing Algorithm for Recent HIV Seroconversion; EIA: Enzyme Immunoassay; Alg: Algorithm; SHCS: Swiss HIV Cohort Study; ZPHI: Zurich Primary HIV Infection Study; WB: Western Blot; Inno-Lia: Inno-Lia™ HIV I/II Score assay; EDI: Estimated Date of Infection; ARS: Acute Retroviral Syndrome; ART: Antiretroviral Treatment; SFOPH: Swiss Federal Office of Public Health; SNCR: Swiss National Center for Retroviruses; IIR: Incident Infection Rate (proportion of incident infections among new HIV-diagnoses).

## Competing interests

The authors declare that no competing interests exist.

Although the study was funded partially by Innogenetics, the decision to conduct the study rested solely with the Swiss HIV Cohort Study (SHCS). The support of Innogenetics was restricted to making possible the testing of samples in the participating centers. All other costs were covered by the Swiss National Science Foundation, the Swiss HIV Cohort Research Foundation and the Swiss Federal Office of Public Health, the public funder of the Swiss National Center for Retroviruses. None of the authors received -- or will receive -- any personal or institutional benefits of any kind.

## Authors' contributions

JS, LRB, SR, PB, MG, CA, SY, TK, HFG and MR designed the study. FSA, MR, HFG and JS selected the patients. LRB, SR, PB, MG, TK, CA, GM, CN, SY, SP, DS and MB conducted the testing and provided the Inno-Lia results. JS and AUS performed the statistical analysis. MDG provided the HIV notification data. JS developed the algorithms, evaluated the data, produced tables and figures and wrote the first draft of the manuscript. All authors critically reviewed and improved the manuscript, and all approved the submitted version.

## Endnote

*This study has been conducted partially in the framework of the Swiss HIV Cohort Study, supported by the Swiss National Science Foundation (grant # 33CS30_134277).

The members of the Swiss HIV Cohort Study are Barth J, Battegay M, Bernasconi E, Böni J, Bucher HC, Bürgisser P, Burton-Jeangros C, Calmy A, Cavassini M, Dubs R, Egger M, Elzi L, Fehr J, Fischer M, Flepp M, Francioli P (President of the SHCS), Furrer H (Chairman of the Clinical and Laboratory Committee), Fux CA, Gorgievski M, Günthard H (Chairman of the Scientific Board), Hasse B, Hirsch HH, Hirschel B, Hösli I, Kahlert C, Kaiser L, Keiser O, Kind C, Klimkait T, Kovari H, Ledergerber B, Martinetti G, Martinez de Tejada B, Müller N, Nadal D, Pantaleo G, Rauch A, Regenass S, Rickenbach M (Head of Data Center), Rudin C (Chairman of the Mother & Child Substudy), Schmid P, Schultze D, Schöni-Affolter F, Schüpbach J, Speck R, Taffé P, Telenti A, Trkola A, Vernazza P, von Wyl V, Weber R, Yerly S.

## Pre-publication history

The pre-publication history for this paper can be accessed here:

http://www.biomedcentral.com/1471-2334/12/88/prepub

## Supplementary Material

Additional file 1 **Changes in the incident infection rate (IIR) among four annual cohorts of HIV-1 notifications (PDF 77 kb)**. Click here for file
