# Supplementary material for: Diagnostic performance of line-immunoassay based algorithms for incident HIV-1 infection
Source: BMC Infect Dis. 2012 Apr 12;12:88. doi: 10.1186/1471-2334-12-88 (PMC3362747; doi:10.1186/1471-2334-12-88)
Supplement: Additional file 1 — Changes in the incident infection rate (IIR) among four annual cohorts of HIV-1 notifications (PDF 77 kb). [file 1471-2334-12-88-S1.PDF]

Additional File 1. Changes in the incident infection rate (IIR) among four annual cohorts of HIV-1 notifications

A. Calculations based on the adjusted, but not weighted sensitivities S1

	Performance		2005/6 (Baseline); N = 748				2008; N = 667					2009; N = 578					2010; N = 602				
ALG #	Adjusted Sensitivity S1 (%)	Specificity (%)	N ruled older	N ruled incident	N estimated incident	IIR	N ruled older	N ruled incident	N estimated incident	IIR	IIR, in % of baseline	N ruled older	N ruled incident	N estimated incident	IIR	IIR, in % of baseline	N ruled older	N ruled incident	N estimated incident	IIR	IIR, in % of baseline
15.1	23.66	95.14	611	137	535	0.716	524	143	588	0.882	123.2	461	117	473	0.818	114.3	494	108	419	0.696	97.2
15	23.85	94.32	608	140	537	0.718	523	144	584	0.876	122.0	458	120	480	0.830	115.7	489	113	434	0.721	100.4
11.2	23.60	94.05	621	127	467	0.625	531	136	546	0.818	130.9	459	119	479	0.829	132.7	498	104	386	0.642	102.7
7	20.34	98.38	656	92	427	0.570	558	109	524	0.786	137.9	497	81	383	0.662	116.1	531	71	327	0.543	95.3
13	22.68	95.00	625	123	484	0.647	538	129	541	0.811	125.3	474	104	425	0.735	113.5	505	97	378	0.629	97.1
12.1	23.66	93.38	619	129	466	0.624	528	139	557	0.834	133.8	458	120	480	0.830	133.1	495	107	394	0.655	105.0
9	19.91	98.38	660	88	415	0.555	560	107	526	0.788	142.2	504	74	353	0.611	110.2	535	67	313	0.520	93.7
4.1	24.54	91.89	597	151	550	0.735	522	145	553	0.830	112.9	450	128	494	0.854	116.2	474	128	482	0.801	108.9
11.1	23.60	93.37	620	128	462	0.618	531	136	541	0.811	131.3	458	120	481	0.833	134.8	496	106	389	0.647	104.7
8.1	20.92	96.76	654	94	395	0.528	554	113	517	0.775	146.9	488	90	403	0.698	132.2	525	77	325	0.540	102.4
mean	**22.68**	**95.07**		***mean***	**474**	**0.633**		***mean***	**548**	**0.821**	**130.6**		***mean***	**445**	**0.770**	**121.9**		***mean***	**385**	**0.639**	**100.7**
SD	1.66	2.17		*SD*	54	0.072		*SD*	24	0.036	10.2		*SD*	50	0.087	9.9		*SD*	53	0.088	4.9
lower limit 95%CI	*21.65*	*93.72*		*lower limit 95%CI*	*441*	*0.589*		*lower limit 95%CI*	*533*	*0.799*	*124.3*		*lower limit 95%CI*	*414*	*0.716*	*115.7*		*lower limit 95%CI*	*352*	*0.585*	*97.7*
upper limit 95%CI	*23.70*	*96.41*		*upper limit 95%CI*	*507*	*0.678*		*upper limit 95%CI*	*563*	*0.843*	*136.9*		*upper limit 95%CI*	*476*	*0.824*	*128.0*		*upper limit 95%CI*	*418*	*0.694*	*103.7*

B. Calculations based on the adjusted and weighted sensitivities S2

	Performance		2005/6 (Baseline); N = 748				2008; N = 667					2009; N = 578					2010; N = 602				
ALG #	Adjusted Sensitivity S2 (%)	Specificity (%)	N ruled older	N ruled incident	N estimated incident	IIR	N ruled older	N ruled incident	N estimated incident	IIR	IIR, in % of baseline	N ruled older	N ruled incident	N estimated incident	IIR	IIR, in % of baseline	N ruled older	N ruled incident	N estimated incident	IIR	IIR, in % of baseline
15.1	49.89	95.14	611	137	224	0.299	524	143	246	0.368	123.2	461	117	197	0.342	114.3	494	108	175	0.290	97.2
15	50.35	94.32	608	140	218	0.292	523	144	238	0.356	122.0	458	120	195	0.338	115.7	489	113	176	0.293	100.4
11.2	49.74	94.05	621	127	188	0.252	531	136	220	0.330	130.9	459	119	193	0.334	132.7	498	104	156	0.259	102.7
7	43.54	98.38	656	92	191	0.255	558	109	234	0.351	137.9	497	81	171	0.296	116.1	531	71	146	0.243	95.3
13	48.18	95.00	625	123	198	0.265	538	129	221	0.332	125.3	474	104	174	0.301	113.5	505	97	155	0.257	97.1
12.1	49.89	93.38	619	129	184	0.246	528	139	219	0.329	133.8	458	120	189	0.327	133.1	495	107	155	0.258	105.0
9	42.48	98.38	660	88	186	0.248	560	107	235	0.353	142.2	504	74	158	0.274	110.2	535	67	140	0.233	93.7
4.1	51.31	91.89	597	151	209	0.280	522	145	210	0.315	112.9	450	128	188	0.325	116.2	474	128	183	0.304	108.9
11.1	49.74	93.37	620	128	182	0.243	531	136	213	0.319	131.3	458	120	189	0.328	134.8	496	106	153	0.255	104.7
8.1	44.20	96.76	654	94	170	0.228	554	113	223	0.335	146.9	488	90	174	0.301	132.2	525	77	140	0.233	102.4
mean	**47.93**	**95.07**		***mean***	**195**	**0.261**		***mean***	**226**	**0.339**	**130.6**		***mean***	**183**	**0.316**	**121.9**		***mean***	**158**	**0.263**	**100.7**
SD	3.24	2.17		*SD*	17	0.023		*SD*	12	0.017	10.2		*SD*	13	0.022	9.9		*SD*	15	0.025	4.9
lower limit 95%CI	*45.92*	*93.72*		*lower limit 95%CI*	*184*	*0.247*		*lower limit 95%CI*	*219*	*0.328*	*124.3*		*lower limit 95%CI*	*175*	*0.303*	*115.7*		*lower limit 95%CI*	*149*	*0.247*	*97.7*
upper limit 95%CI	*49.94*	*96.41*		*upper limit 95%CI*	*206*	*0.275*		*upper limit 95%CI*	*233*	*0.350*	*136.9*		*upper limit 95%CI*	*191*	*0.330*	*128.0*		*upper limit 95%CI*	*167*	*0.278*	*103.7*

C. Calculations based on the adjusted and weighted sensitivities S3

	Performance		2005/6 (Baseline); N = 748				2008; N = 667					2009; N = 578					2010; N = 602				
ALG #	Adjusted Sensitivity S3 (%)	Specificity (%)	N ruled older	N ruled incident	N estimated incident	IIR	N ruled older	N ruled incident	N estimated incident	IIR	IIR, in % of baseline	N ruled older	N ruled incident	N estimated incident	IIR	IIR, in % of baseline	N ruled older	N ruled incident	N estimated incident	IIR	IIR, in % of baseline
15.1	34.17	95.14	611	137	343	0.459	524	143	377	0.566	123.2	461	117	303	0.525	114.3	494	108	269	0.446	97.2
15	34.45	94.32	608	140	339	0.453	523	144	369	0.553	122.0	458	120	303	0.524	115.7	489	113	274	0.455	100.4
11.2	34.07	94.05	621	127	293	0.392	531	136	342	0.513	130.9	459	119	301	0.521	132.7	498	104	242	0.403	102.7
7	29.52	98.38	656	92	286	0.383	558	109	352	0.528	137.9	497	81	257	0.444	116.1	531	71	220	0.365	95.3
13	32.83	95.00	625	123	308	0.411	538	129	344	0.515	125.3	474	104	270	0.467	113.5	505	97	240	0.399	97.1
12.1	34.17	93.38	619	129	289	0.386	528	139	344	0.516	133.8	458	120	297	0.513	133.1	495	107	244	0.405	105.0
9	28.86	98.38	660	88	279	0.372	560	107	353	0.529	142.2	504	74	237	0.411	110.2	535	67	210	0.349	93.7
4.1	35.34	91.89	597	151	332	0.444	522	145	334	0.500	112.9	450	128	298	0.515	116.2	474	128	291	0.483	108.9
11.1	34.07	93.37	620	128	286	0.382	531	136	334	0.501	131.3	458	120	298	0.515	134.8	496	106	241	0.400	104.7
8.1	30.22	96.76	654	94	259	0.346	554	113	339	0.508	146.9	488	90	264	0.457	132.2	525	77	213	0.354	102.4
mean	**32.77**	**95.07**		***mean***	**301**	**0.403**		***mean***	**349**	**0.523**	**130.6**		***mean***	**283**	**0.489**	**121.9**		***mean***	**244**	**0.406**	**100.7**
SD	2.34	2.17		*SD*	28	0.038		*SD*	14	0.022	10.2		*SD*	24	0.041	9.9		*SD*	27	0.044	4.9
lower limit 95%CI	*31.32*	*93.72*		*lower limit 95%CI*	*284*	*0.379*		*lower limit 95%CI*	*340*	*0.510*	*124.3*		*lower limit 95%CI*	*268*	*0.464*	*115.7*		*lower limit 95%CI*	*228*	*0.378*	*97.7*
upper limit 95%CI	*34.22*	*96.41*		*upper limit 95%CI*	*319*	*0.426*		*upper limit 95%CI*	*358*	*0.536*	*136.9*		*upper limit 95%CI*	*297*	*0.515*	*128.0*		*upper limit 95%CI*	*261*	*0.433*	*103.7*
